# The Keystone State Takes an Important Step

**Published:** 1897-08

**Authors:** 


### THE KEYSTONE STATE TAKES AN IMPORTANT STEP.

PENNSYLVANIA'S Legislature has just appropriated the sum of fifteen thousand dollars for use in the investigation of contagious and infectious diseases of domestic animals. We believe this is the second State to move in this special direction. Nebraska led in this movement, and while many other States have yearly appropriated money to control these diseases, none save those mentioned have equipped themselves for original investigation and appropriated money for this special work. Pennsylvania's representatives are to be congratulated on this important step, for it means much to those engaged in the live-stock industry, and whose burdens have been specially heavy to bear in recent years. It means much to her people in the better control of a purer and more wholesome meat- and milk-supply. Specially are the veterinary profession to be grateful to State Veterinarian Pearson, whose indefatigable labors have brought forth this result under the most adverse circumstances that have ever confronted new legislation of any kind that demanded State funds for its enforcement. Early and late he labored among those whose interests were imperilled, and in an educational manner impressed all who had at heart the agricultural and live-stock interests the importance of the work, and that the Keystone State should be among those that lead in this interest, as she has done in so many other directions. Governor Hastings, fully concurring in the desire to render these interests better protection and stronger safeguards, gave the bill his approval, and he thus renders to the people of the whole State a benefit that cannot be estimated in a monetary aspect.

